# The Hidden One: What We Know About Bitter Taste Receptor 39

**DOI:** 10.3389/fendo.2022.854718

**Published:** 2022-03-08

**Authors:** Florijan Jalševac, Ximena Terra, Esther Rodríguez-Gallego, Raúl Beltran-Debón, Maria Teresa Blay, Montserrat Pinent, Anna Ardévol

**Affiliations:** MoBioFood Research Group, Departament de Bioquímica i Biotecnologia, Universitat Rovira i Virgili, Tarragona, Spain

**Keywords:** TAS2R39, bitter taste, catechin, GPCR, food intake, respiratory system, TAS2R39 agonist, TAS2R39 antagonist

## Abstract

Over thousands of years of evolution, animals have developed many ways to protect themselves. One of the most protective ways to avoid disease is to prevent the absorption of harmful components. This protective function is a basic role of bitter taste receptors (TAS2Rs), a G protein-coupled receptor family, whose presence in extraoral tissues has intrigued many researchers. In humans, there are 25 TAS2Rs, and although we know a great deal about some of them, others are still shrouded in mystery. One in this latter category is bitter taste receptor 39 (TAS2R39). Besides the oral cavity, it has also been found in the gastrointestinal tract and the respiratory, nervous and reproductive systems. TAS2R39 is a relatively non-selective receptor, which means that it can be activated by a range of mostly plant-derived compounds such as theaflavins, catechins and isoflavones. On the other hand, few antagonists for this receptor are available, since only some flavones have antagonistic properties (all of them detailed in the document). The primary role of TAS2R39 is to sense the bitter components of food and protect the organism from harmful compounds. There is also some indication that this bitter taste receptor regulates enterohormones and in turn, regulates food intake. In the respiratory system, it may be involved in the congestion process of allergic rhinitis and may stimulate inflammatory cytokines. However, more thorough research is needed to determine the precise role of TAS2R39 in these and other tissues.

## Introduction

Humans have five primary tastes: bitter, sweet, sour, salty, and umami (1), which are identified as membrane receptors. These receptors enable animals to analyze the chemical structure of food as soon as it hits the taste buds located on their tongue before any harmful components are digested. Although on the surface the role of these receptors may look the same, they are anything but alike. Though ubiquitous to us all, sensing salty taste remained a mystery until recently when researchers identified specialized cells that recognize this taste through the sodium ion channel (2). Other types of ion channels are used to detect the sour components of food (3), while the other three tastes are mediated differently, i.e. through specialized G protein-coupled receptors (GPCRs) known as taste receptors (TASRs) (4). Although these receptors sense different compounds, they share several structural and functional attributes. These glycoprotein receptors consist of seven transmembrane α-helices. Upon binding of a ligand, interaction with heterotrimeric G proteins occurs, which activates the propagation of signaling cascade in the cell (5). To identify umami, sweet and bitter tastes, two families of GPCRs have been identified: TAS1R and TAS2R. The first family of receptors contains three members: TAS1R1, TAS1R2 and TAS1R3 (6). This family is responsible for detecting both sweet and umami tastes. This is done by dimerizing different members into one receptor: the TAS1R1-TAS1R3 heterodimer is responsible for umami, while TAS1R2-TAS1R3 complex is responsible for sweet (7). On the other hand, the TAS2R family is made up of a much more diverse group of receptors, which in humans consists of 25 functional genes, as well as 8 pseudogenes, though the number varies greatly from species to species (8, 9). Researchers have also discovered that although we possess a relatively low number of bitter taste receptors, we are able to detect as bitter thousands of compounds from a wide range of families. We can categorize bitter receptors into four groups based on their specificity to ligands: receptors that detect a broad spectrum of ligands; more selective receptors with just a few ligands; receptors somewhere between these two groups; and receptors that recognize specific chemical motifs (10). One member of the group that detects a moderate number of bitter compounds is TAS2R39, which is the focal point of this review.

## TAS2R39 Expression in the Body

TAS2R39 is encoded by the *TAS2R39* gene, which is located on chromosome 7 (7q34). Interestingly, this gene sequence contains no intron, so it belongs to a small group of genes that are known as intron-less or single-exon genes (11). This bitter taste receptor is still relatively unknown, since the first ligands for it were discovered just over a decade ago (4). Another area where more concrete information is lacking are the locations where it is expressed. As with other members of the bitter-taste receptor family, TAS2R39 is expressed in the oral tissue. The little information available about its expression indicates that it is one of the TAS2Rs that are expressed in the lowest quantities (12). The extraoral presence of bitter taste receptors has been well documented. These can be found in several types of tissues, including the brain, the respiratory system, the cardiac system, and even the reproductive system (6, 13–15). However, discrepancies exist among these receptors since some are more well-known, while others remain unexplored. Studies exploring the expression of the *TAS2R39* gene have identified TAS2R39 in the colon (16), bronchi (17), nasal mucosa (14), arteries (18), and skin (19). Additionally, cell lines used in research have been identified as expressing this gene, including the HuTu-80 and NCI-H716 intestinal cells (16), the hTERT-HM myometrial cells (20), and lung macrophages (21), while the protein has been detected in the cells of choroid plexus of the nervous system (22). Further data are constantly being added to databases such as the Human Protein Atlas, while new locations for TAS2R39 expression are being discovered, including the pancreas and spleen, and even the brain, testes and ovaries (11, 23). Although TAS2R39 appears to be widely present in human tissues, it is important to note that in all these tissues its gene expression is low and, that, as is reflected by the relatively little knowledge we have about this bitter taste receptor, detecting it is a challenge.

## Structure and Signal Transduction

TAS2R39 is a 338 amino acid long protein that, as we mentioned earlier, has seven transmembrane domains (numbered from I to VII). Based on computer analysis using modelling software, a structure-based pharmacophore model was created (24). This model suggests that the binding pocket of a TAS2R39 ligand is located extracellularly between transmembrane helices III, V, VI and VII, with a hydrophobic interaction between the ligand and receptor that covers most of the interaction area. Additionally, hydrogen bond acceptors and donors contribute to the binding and, potentially, π-π aromatic influences may also occur in the binding pocket. The structural differences between agonists and antagonists have also been inspected. Although at first sight they are quite similar, two key differences have been identified: as well as lacking a hydrogen donor, antagonists display stereochemical flexibility, which fills the binding pocket and, in this way, prevent a conformational change in the receptor upon its activation (24). Another study compared ligands that activate TAS2R14 and TAS2R39 by analyzing the structure-activity relationship. In that study it was observed that glycosylation had an inhibiting effect on TAS2R14, while activation of the TAS2R39 was preserved (though a higher concentration of agonists was needed to activate the receptor). The compounds tested were members of the isoflavonoid group. Alteration of the C-ring skeletal structure of these compounds did not hinder the activation of these two receptors, though their potency and efficacy may be affected. Also inspected was the effect of different substituents on the binding properties. It was found that, for a compound to be a TAS2R39 ligand, substitutes, preferably a hydroxy group, are obligatory (25).

**Figure 1:**
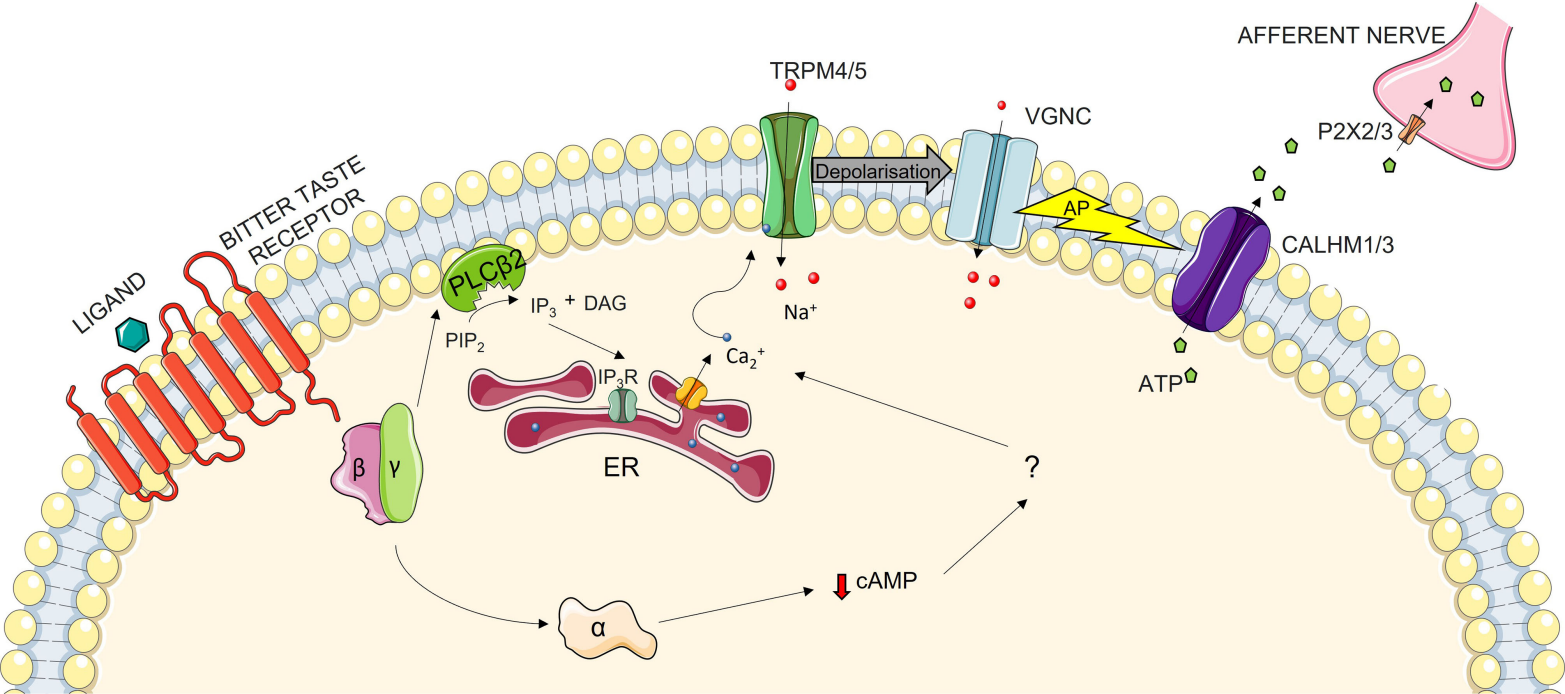
The Bitter Taste Receptor Signaling Pathway: when the ligand binds to the TAS2R, a conformational change is induced. This in turn triggers the dissociation of the α-subunit of gustducin from the β- and γ-subunits. This dissociation of subunits signifies two divergent pathways of signal transduction. In the first of these, β- and γ-subunits activate the β2 isoform of phospholipase C (PLCβ2), which cleaves phosphatidylinositol 4,5-bisphosphate (PIP_2_) into diacyl glycerol (DAG) and inositol 1,4,5-trisphosphate (IP_3_). IP_3_ then travels to Endoplasmic Reticulum (ER), where it binds to its receptor (IP_3_R), leading to the secretion of Ca_2_^+^ from the ER into the cytoplasm. This increase in intracellular Ca_2_^+^ levels leads to the activation of sodium-selective transmembrane transporters TRPM4 and 5, thereby depolarizing the cellular membrane. Depolarization activates voltage-gated sodium channels (VGNC), thus hastening depolarization. When the action potential (AP) is reached, the calcium homeostasis modulator 1 and 3 (CALHM1/3) channel and pannexin 1 channels are activated, which leads to the transportation of ATP from cytoplasm to the intercellular space. Through P2X ionotropic purinergic receptors 2 and 3 (P2X2/P2X3), ATP is taken into the afferent nerve, thereby propagating the signal further down. The other pathway occurs through the α-subunit of gustducin, but the exact mechanism of signal propagation is unknown. It is postulated that α-gustducin lowers the level of cAMP by activating its hydrolysation though phosphodiesterase (PDE), but the next steps are still unknown. The lowering of cAMP may lead to a decrease in the levels of cNMPs intracellularly, which may regulate protein kinases and in turn regulate the ion activity in the cell. It is also possible that cNMP directly regulates cNMP-gated ion channels, thereby depolarizing the membrane and eliciting the release of the neurotransmitter.

All elements of the signaling cascade of the TAS2R signaling pathway remain unknown. However, researchers have discovered the importance of gustducin as a member of the G-coupled protein mechanism (26, 27). This is even more evident from the fact that TAS2Rs are expressed in cells containing gustducin (28, 29). The perception of taste starts on the tongue in the taste organ, i.e. the taste bud (30). Located throughout the oral cavity, these buds contain between 50 and 100 taste cells (31). Each of the five basic tastes is sensed by specific cells. Cells that detect bitter taste are called type-II cells, which can also sense sweet and umami tastes. Which taste they detect depends on the receptor that is expressed (32). Although it has not been confirmed, we can postulate that the signaling pathway in the oral cavity is similar in the extraoral locations. When a bitter ligand binds to the receptor, a conformational change is induced. This causes the dissociation of α-gustducin from the β and γ subunits of the G protein-coupled unit. The β and γ subunits of this protein then activate the β2 isoform of phospholipase C (PLCβ2), which in turn leads to the synthesis of inositol 1,4,5-triphosphate (IP3) and diacylglycerol (DAG). IP3 binds to IP3 receptor, resulting in the release of Ca^2+^ from the endoplasmic reticulum. This change in the intracellular concentration of ions induces the activation of membrane ion channels, which transport Na^+^ into the cells, thereby depolarizing the membrane. Once action potential is reached, neurotransmitter ATP is released from the cells, with the signal propagating forwards through afferent nerves (33, 34). However, this is not the only way the signaling pathway is activated. Upon ligand binding, another pathway is activated: the α-gustducin subunit stimulates taste phosphodiesterase (PDE), which hydrolyses cAMP. What happens then is not known but some theories suggest that this may disinhibit cyclic nucleotide-inhibited channels and in turn elevate the intracellular levels of Ca^2+^, which again results in the exocytosis of neurotransmitters (**Figure 1**) (33, 35).

## Ligands

For a long time, TAS2R39 was considered an orphan receptor, i.e. there were no known ligands that activated it. Recently, however, interest in this receptor has increased and, in 2009, its first ligands were discovered (10). These first ligands discovered were agonists, and most compounds were extracted or derived from plants. Various groups of TAS2R39 ligands also appeared, some of which were more specific, while others also bound to other bitter taste receptors. More studies exploring possible ligands, the most prevalent source of which were again plants, were then conducted. With theaflavins extracted from black tea, TAS2R39 shows a preference for theaflavin and theaflavin-3,3-O’-digalatte, while theaflavin-3-O’-galatte also activated TAS2R14 (36). Catechins derived from green tea are another group of ligands for this bitter taste receptor, with epicatechin gallate and epigallocatechin gallate demonstrating relatively strong affinity to TAS2R39, and epicatechin and epigallocatechin displaying lower affinity. These data show that the galloyl group creates important bonds for the ligand-receptor interaction. Note that these catechins also bind to other bitter receptors, and the only one that seems to be selective for the TAS2R39 is epigallocatechin (37). Soybean is another plant from which such compounds can be extracted. Genistein, a predominant isoflavone in soybeans, activated both TAS2R39 and TAS2R14, which is becoming TAS2R39’s closest relative in terms of the ligands they both bind to. However, glucosylated forms of genistein do not activate TAS2R14, though they did activate TAS2R39. Further experiments with similar isoflavones were conducted and 15 other TAS2R39 agonists were discovered, with acetylgenistin, genistin, glycitin and malonyl genistin being specific to TAS2R39 (25). A more comprehensive study was conducted in which 67 flavonoid and isoflavonoid compounds were found to be ligands for this receptor, with new specific ligands being acacetin, 5,2′-dihydroxyflavone, gardenin A, genkwanin gossypetin, 6-methoxyflavonol and 4′-hydroxyflavanone (38). A recent study also noted the activation of this bitter taste receptor by vanillin, a major component of vanilla widely used as a flavouring agent (39).

Small molecules are not the only ones that can activate this bitter taste receptor, as some peptides have been also identified as TAS2R39 agonists. Amino acids, especially those exhibiting more hydrophobic properties, show greater affinity to the receptor, since hydrophobicity is one of the major factors of ligand-bitter taste receptor interaction (40). A broad study testing all proteinogenic amino acids on all bitter taste receptors uncovered more details about the amino acid activation of bitter taste receptors. It was discovered that the most sensitive bitter taste receptors for amino acids are TAS2R1, TAS2R4, and TAS2R39, since these were activated by phenylalanine (Phe) and tryptophan (Trp). Curiously, it was observed that TAS2R39 was activated by the D-conformation amino acids, such as D-Trp. Note that these agonists expressed low levels of receptor activation. In the next stage of the study, dipeptides and tripeptides from the previously mentioned amino acids were examined. Interestingly, TAS2R39 was activated by Trp-Trp and Leu-Trp dipeptides but not by other Trp-containing combinations (like Trp-Leu), which indicates that the interaction between these kinds of ligands and receptors cannot be described solely by the presence or position of the activating amino acid in a dipeptide. Of the tested tripeptides, Trp-Trp-Trp and Leu-Leu-Leu both activated TAS2R39 (41). Finally, larger peptides found in the cheese-maturation process also activated TAS2R39 as well as TAS2R1.

Although most ligands now discovered act as agonists of the TAS2R39, some studies also identified antagonists for this receptor (42). Like many agonists, specific blockers for these bitter taste receptors come from the flavanone group of compounds. Two of the 14 screened flavanones 6,3’-dimethoxyflavanone and 4’-fluoro-6-methoxyflavanone showed clear inhibiting tendencies against TAS2R39, while 6-methoxyflavanone displayed a less inhibiting tendency. Researchers also tested the specificity of these ligands to TAS2R39 by comparing this bitter taste receptor with the other receptor that binds many of the flavonoids, i.e. TAS2R14. Here an inhibition was also observed but to a lesser extent than with TAS2R39. Structural analysis of these inhibitors showed that for a ligand to act as an antagonist on this bitter taste receptor, a methoxy group on position 6 of the A ring is mandatory, as is the absence of a double bond in the C ring of the structure (42). All currently known TAS2R39 exclusive receptor ligands can be found in the **Table 1**, while all known TAS2R39 ligands are listed in the **Supplementary Table S1**.

**Table 1:** Currently described as exclusive hTAS2R39 ligands.

Ligand	Effective concentration (μM)	EC50 (μM)	Source
AGONISTS			
4’-hydroxyflavone	500	nd	(38)
5, 2’-dihydroxyflavone	500	nd	(38)
5-hydroxyflavone	500	nd	(38)
Acetylgenistin	125	nd	(38)
Epigallocatechin*	nd	395.5	(37, 43)
Fisetin	1	nd	(38)
Genistin	500	nd	(25)
Genkwanin	500	nd	(38)
Glycitin	500	nd	(25)
Gossypetin	250	388	(38)
Malonylgenistin	500	nd	(25)
Paracetamol*	3000	nd	(10)
Prolylarginine	10000	nd	(44)
Theaflavin*	nd	2.79	(36)
Theaflavin- 3, 3’-O-digallate*	nd	1.55	(36)
Tricetin	250	nd	(38)
ANTAGONISTS			
4’-fluoro-6-methoxyflavanone	nd	102	(42)
6, 3’-dimethoxyflavanone	nd	407	(42)

*These are the ligands that have been tested on all 25 human bitter taste receptors and have activated only TAS2R39 receptor. nd, not determined.

What is evident from the current data is that since studies of these bitter taste receptors are still ongoing and the first specific ligands were discovered only a decade ago, we still have much to learn about the interactions between the receptor and its ligands and about how to define more ligands, specific agonists, and antagonists. More specific ligands on TAS2R39 could help us discover more specific properties of this bitter taste receptor and clarify its physiological and potential pathophysiological roles.

## Physiological Role

Animal models, especially rat and mouse, are key to understanding protein function in humans when there are orthologues for the protein under study. It is not surprising, therefore, that, apart from in humans, our knowledge of bitter taste receptors in these two species is the broadest. Roughly 35 functional genes have been discovered in mice (45) and roughly the same number have been discovered in rats (46). However, the mystery surrounding bitter taste receptors in these animals remains. Although some have been deorphanized (47), there is much more that we do not know. We can see that – like in humans – rats and mice possess different groups of TAS2Rs, with some responding to a broad range of ligands and others being tuned more specifically. Also, just as we observed the expression of taste receptors in a myriad of human tissues, the same is true for these rodents: bitter receptors were detected, for example, in the nasal mucosa (14), vascular system (48), and intestinal system (49) of rats, and probably also appear in many other tissues.

Although their name appears to suggest that they have a simple assignment, bitter taste receptors are involved in numerous biological processes and are therefore an important component of homeostasis. As stated earlier, their primary function is, in conjunction with other taste receptors, to sense the nutritional constituents of ingested food, but they also have a specific role in sensing the presence of toxic components such as plant alkaloids to prevent their ingestion before they harm the organism (15). However, not all bitter compounds are dangerous and some are beneficial to our health (50). Moreover, many common foods and beverages, such as cocoa beans, coffee and beer, are bitter, and we still enjoy their taste. As well as taste sensing, numerous studies have analyzed the function of this receptor in other tissues and systems. There is reliable evidence that bitter taste receptors are involved in glucose homeostasis (51). A possible role of various bitter taste receptors (including the ortholog to human TAS2R39, TAS2R139) in the control of food intake has been studied in the gastrointestinal system of rats (52). Bitter compounds and, in turn, TAS2Rs, have been linked with controlling the secretion of ghrelin, a hormone known as the hunger hormone, which, when released, increases food intake (52). Protein YY (PYY), glucagon-like peptide 1 (GLP-1) and cholecystokinin (CCK) have also been identified as enterohormones that control the appetite (53) and can be influenced by bitter compounds. It was discovered that by activating certain TAS2Rs, such as TAS2R5, GLP-1 secretion can be increased, thus leading to a decrease in food intake in rats. However, when TAS2R39 was targeted specifically, PYY secretion increased with no influence on food intake. Also, when a combination of ligands that bind to other bitter taste receptors but are preferential to TAS2R39 was tested, an increase in food intake was noted (54).

Another area where bitter taste receptors are important is the respiratory system. Their presence in this system has been well documented (55), while it is suggested that taste receptors there are important components of the innate immune system. TAS2R39 has been detected in the human bronchi but it is unlikely to be involved in bronchial relaxation since the ligands that bind to it produced no effects (17). However, once non-specific agonists were used, bronchial relaxation was observed, so the role of other bitter taste receptors cannot be ignored. The importance of TAS2Rs in the respiratory system came out of a study that inspected nasal mucosa in patients suffering from allergies. Both healthy individuals and allergic rhinitis patients were included in the study, which showed that bitter taste receptors, including TAS2R39, are ubiquitously present in nasal tissue and that their expression is increased in allergic patients. Further experiments revealed that TAS2R39 expression in this type of tissue increases when stimulated by certain cytokines, namely IL-3, IL-5, IL-10 and TGF-β. Whereas interleukins induced the expression of several bitter taste receptors, TGF-β up-regulated only TAS2R39 (14). Moreover, stimulation of the nasal mucosa by various bitter compounds triggered constriction of the mucosa in patients, while in rats, vascular constriction of the mucosa was observed. This suggests that bitter taste receptors may have an important role in constriction of the nasal mucosa, thus improving congestion and controlling allergic rhinitis. However, as some bitter taste receptors (including TAS2R39) were up-regulated by inflammatory cytokines, it may be that these receptors also participate in the pathogenesis of inflammatory diseases. These conflicting results reinforce the need for more thorough studies to decipher the role of these receptors in the homeostasis of organisms.

## Conclusions

As this is a fairly novel topic of research, the significance of TAS2R39 is yet to be determined. Though present in extraoral locations, it is yet to be fully determined in which specific tissues and at what levels it is present. A more detailed future analysis of the signaling pathway could enable us to better understand its role in extraoral tissues and in turn make it easier to modulate it. Primary research exploring its role in the control of food intake is promising, though more concrete studies are needed to decipher its role in the control of satiety. Its role in the respiratory system also remains unclear since a possible preventive role in congestion seems to be at odds with its possible pro-inflammatory effect. These discrepancies with regard to TAS2R39 show us that without further research, this receptor will remain the hidden one.

## Author Contributions

AA and MP conceived the idea and drafted a proposal. FJ reviewed the literature and scripted the basis of the manuscript. ER-G and RB-D elaborated the figures and tables. MB and XT critically reviewed the manuscript. All the authors reviewed the manuscript and approved the final version.

## Funding

This work was supported the Proyecto AGL2017-83477-R financiado por MCIN/AEI/10.13039/501100011033/FEDER “Una manera de hacer Europa” This project has received funding from the European Union’s Horizon 2020 research and innovation programme under the Marie Skłodowska Curie grant agreement No 945413 and from the Universitat Rovira i Virgili (URV). MP and XT are Serra Húnter fellows. The funding providers had no role in the design, analysis or writing of this article.

## Author Disclaimer

The authors declare that this work reflects only their view and that the European Research Executive Agency is not responsible for any use that may be made of the information it contains.

## Conflict of Interest

The authors declare that the research was conducted in the absence of any commercial or financial relationships that could be construed as a potential conflict of interest.

## Publisher’s Note

All claims expressed in this article are solely those of the authors and do not necessarily represent those of their affiliated organizations, or those of the publisher, the editors and the reviewers. Any product that may be evaluated in this article, or claim that may be made by its manufacturer, is not guaranteed or endorsed by the publisher.
