# Supplementary material for: The Hidden One: What We Know About Bitter Taste Receptor 39
Source: Front Endocrinol (Lausanne). 2022 Mar 8;13:854718. doi: 10.3389/fendo.2022.854718 (PMC8957101; doi:10.3389/fendo.2022.854718)
Supplement: Supplementary file 1 [file DataSheet_1.docx]

Supplementary Material


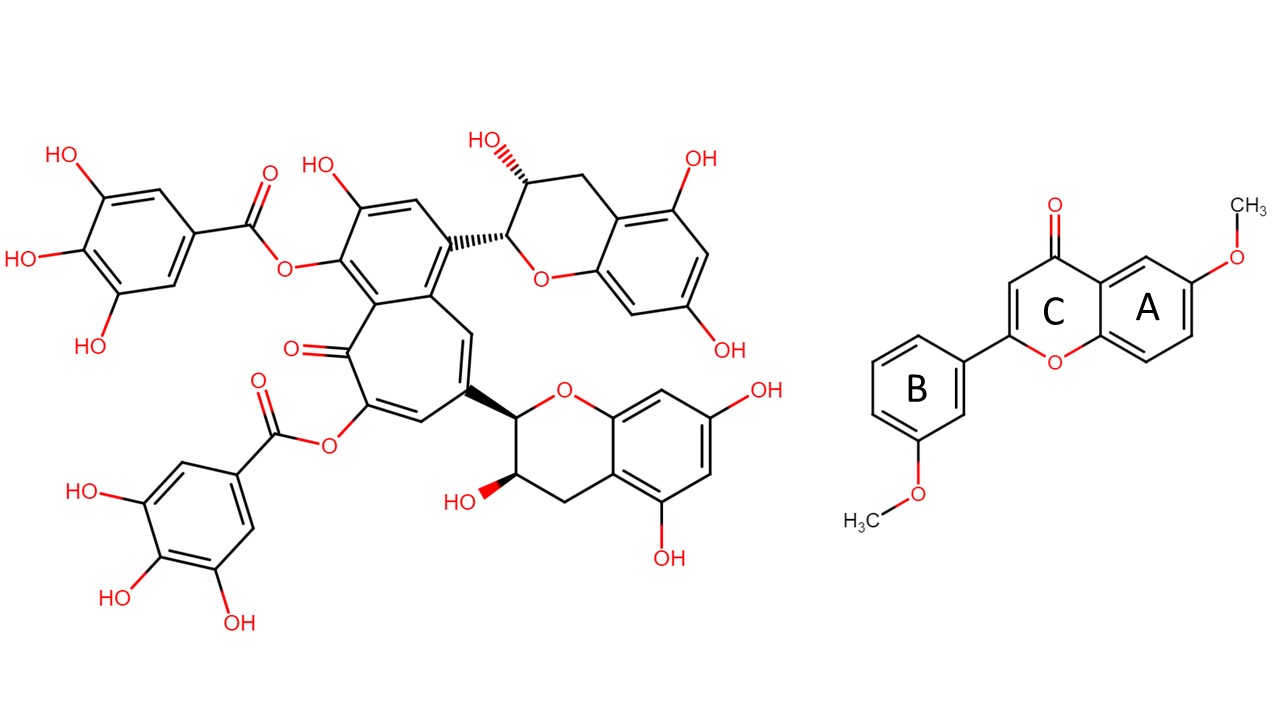


B)

A)

Figure S1: Chemical structures of a TAS2R39 agonist theaflavin-3,3’-digallate (A) and TAS2R39 antagonist 6,3'-dimethoxyflavone (B). In the structure of 6,3'-dimethoxyflavone, commonly applied ring-nomenclature for flavonoids (A-, B-, and C-ring) is shown. Adapted from Chem Space (https://chem-space.com/)

Table S1: All known ligands that activate TAS2R39

| **Ligand** | **Effective concentration on hTAS2R39 (μM)** | **EC50 on TAS2R39 (μM)** | **Specific for TAS2R:** | **Source:** |
| --- | --- | --- | --- | --- |
| AGONISTS | | | | |
| (-)- Epicatechin (EC) | 1000 | 417.1 | 4, 5, 39 | (1), (2) |
| (-)- Epicatechin gallate (ECg) | 32 | 151 | 14, 39 | (1), (2) |
| (-)- Epigallocatechin (EGC) | nd | 395.5 | 39 | (1), (2) |
| (+)- Taxifolin | 125 | nd | 14, 39 | (1) |
| (+/-) Equol | 32 | 55.8 | 14, 39 | (3) |
| 2,2’,4’-trihydroxychalcone | 2 | nd | 14, 39 | (1) |
| 3-(2-hydroxyethyl)-indole | 300 | nd | 4, 39 | (4) |
| 3,2’-dihydroxychalcone | 8 | 53.6 | 14, 39 | (1) |
| 3,6,3’,4’-tetrahydroxyflavone | 2 | nd | 14, 39 | (1) |
| 3,7,4’-trihydroxyflavone | 0.5 | nd | 14, 39 | (1) |
| 4,2’,5’-trihydroxychalcone | 2 | nd | 14, 39 | (1) |
| 4'-hydroxy-7-methoxyflavone | 250 | nd | 14, 39 | (1) |
| 4'-hydroxyflavone | 500 | nd | 39 | (1) |
| 5,2'-dihydroxyflavone | 500 | nd | 39 | (1) |
| 5,4'-dihydroxyflavone | 500 | nd | 14, 39 | (1) |
| 5,7,2'-trihydroxyflavone | 4 | 35.3 | 14, 39 | (1) |
| 5,7'-dimethoxyflavone | 32 | nd | 14, 39 | (1) |
| 5-hydroxyflavone | 500 | nd | 39 | (1) |
| 6,4'-dihydroxyflavone | 500 | nd | 14, 39 | (1) |
| 6,7,4’-trihydroxyisoflavone | 250 | nd | 14, 39 | (3) |
| 6-methoxyluteolin | 8 | 22.9 | 14, 39 | (1) |
| 7,3',4'-trihydroxyflavone | 16 | 141 | 14, 39 | (1) |
| 7,3’,4’-trihydroxyisoflavone | 250 | nd | 14, 39 | (3) |
| 7,4-'dihydroxyflavone | 125 | nd | 14, 39 | (1) |
| 7,8,4’-trihydroxyisoflavone | 63 | 184 | 14, 39 | (3) |
| 7-hydroxyisoflavone | 250 | 315 | 14, 39 | (3) |
| Acetaminophen | 3000 | nd | 39 | (5) |
| Acetylgenistin | 125 | nd | 39 | (3) |
| Amarogentin | 300 | nd | 1, 4, 39, 43, 46, 47, 50 | (5) |
| Apigenin | 1 | nd | 14, 39 | (1) |
| Azathioprine | 1000 | nd | 4, 10, 39, 46 | (5) |
| Biochanin A | 500 | nd | 14, 39 | (3) |
| Butein | 125 | nd | 14, 39 | (1) |
| Chloramphenicol | 1000 | nd | 1, 8, 10, 39, 41, 43, 46 | (5) |
| Chloroquine | 100 | nd | 3, 7, 10, 14, 39 | (5) |
| Chlorpheniramine | 100 | nd | 4, 7, 10, 14, 38, 39, 40, 46 | (5) |
| Chrysin | 16 | nd | 14, 39 | (1) |
| Colchicine | 3000 | nd | 4, 39, 46 | (5) |
| Coumestrol | 250 | nd | 14, 39 | (3) |
| Cyanidin Chloride | 32 | 187 | 14, 39 | (1) |
| Daidzein | 500 | nd | 14, 39 | (3) |
| Datiscetin | 16 | 41.6 | 14, 39 | (1) |
| Denatonium benzoate | 100 | nd | 4, 8, 10, 13, 39, 43, 46, 47 | (5) |
| Denatonium saccharide | nd | nd | 5, 39, 40, 43 | (6) |
| Diphenidol | 100 | nd | 1, 4, 7, 10, 13, 14, 16, 38, 39, 40, 43, 44, 46, 47, 49 | (5) |
| D-tryptophan | 217000 | nd | 4, 39 | (4) |
| Epigallocatechin gallate (EGCg) | 32 | 161 | 39, 43; 14 | (1), (2) |
| Eriodictyol | 16 | 62 | 14, 39 | (1) |
| Eriodictyolchalcone | 16 | 55.5 | 14, 39 | (1) |
| Fisetin | 1 | nd | 39 | (1) |
| Flavone | 8 | 45.9 | 14, 39 | (1) |
| Formomonetin | 500 | nd | 14, 39 | (1) |
| Formononetin | 500 | nd | 14, 39 | (3) |
| Fustin | 250 | nd | 14, 39 | (1) |
| Genistein | 8 | 49.4 | 14, 39 | (3) |
| Genistin | 500 | nd | 39 | (3) |
| Genkwanin | 500 | nd | 39 | (1) |
| Glycitein | 500 | nd | 14, 39 | (3) |
| Glycitin | 500 | nd | 39 | (3) |
| Gossypetin | 250 | 388 | 39 | (1) |
| Herbacetin | 125 | nd | 14, 39 | (1) |
| Hesperitin | 8 | nd | 14, 39 | (1) |
| Homoeriodictyol | 32 | 84.9 | 14, 39 | (1) |
| Isoliquiritigenin | 16 | nd | 14, 39 | (1) |
| Isorhamnetin | 0.12 | nd | 14, 39 | (1) |
| Kaempferol | 14 | nd | 14, 39 | (1) |
| Leu-Trp | 3000 | nd | 1, 4, 39 | (4) |
| Leu-Val-Tyr-Pro-Phe-Pro-Gly-Pro-Ile-His-Asn | 1000 | nd | 1, 39 | (4) |
| Liquiritigenin | 16 | 64.5 | 14, 39 | (1) |
| Luteolin | 0.5 | 7.3 | 14, 39 | (1) |
| Malonylgenistin | nd | 500 | 39 | (3) |
| Morin | 2 | nd | 14, 39 | (1) |
| Myricetin | 1 | nd | 14, 39 | (1) |
| Naringenin | 8 | 32.9 | 14, 39 | (1) |
| Pelargoninidin Chloride | 32 | nd | 14, 39 | (1) |
| Pentagalloylglucose | 3 | 6.6 | 5, 39 | (7) |
| Phe-Phe-Pro-Arg | nd | nd | 8, 39 | (8) |
| Phloretin | 8 | 41.3 | 14, 39 | (1) |
| Pinocembrin | 4 | 48.9 | 14, 39 | (1) |
| Pro-Arg (Prolylarginine) | 10000 | nd | 39 | (8) |
| Pyrocatechin | nd | nd | 1, 14, 39 | (6) |
| Quercetagetin | 2 | nd | 14, 39 | (1) |
| Quinine | 10 | nd | 4, 7, 10, 14, 39,40, 43, 44, 46 | (5) |
| Resveratrol | 63 | 109 | 14, 39 | (1) |
| Scutellarein | 8 | 40.3 | 14, 39 | (1) |
| Silibinin | 8 | 99.2 | 14, 39 | (1) |
| Sucralose | nd | nd | 1, 4, 5, 7, 8, 10, 39, 41, 46 | (6) |
| Sulfuretin | 16 | 48 | 14, 39 | (1) |
| Tenofovir Alafenamide (TAF) | nd | 0.87 | 1,8, 14, 39 | (9) |
| Theaflavin | nd | 2.79 | 39 | (10) |
| Theaflavin-3,3'-O-digallate | nd | 1.55 | 39 | (10) |
| Theaflavin-3'-O-gallate | nd | 0.67 | 3, 14, 39 | (10) |
| Thiamine | 1000 | nd | 1, 39 | (5) |
| Tricetin | 250 | nd | 39 | (1) |
| Trp-Trp | 1000 | 660 | 1, 4, 39 | (4) |
| Trp-Trp | 10000 | nd | 1, 4, 39 | (4) |
| Trp-Trp-Trp | 100 | nd | 1, 4, 14, 39, 46 | (4) |
| Tyr-Pro-Phe-Pro-Gly-Pro-Ile-His-Asn-Ser | 1000 | nd | 1, 39 | (4) |
| Vanilin | nd | 0.87 | 14, 29, 39 | (11) |
| Xanthone | 500 | nd | 14, 39 | (1) |
| ANTAGONISTS | | | | |
| 4'-fluoro-6-methoxyflavanone | nd | 102 | 14, 39 | (12) |
| 6,3'-dimethoxyflavanone | nd | 407 | 14, 39 | (12) |
| 6-methoxyflavanone | nd | 479 | 39 | (12) |
| 6-Methylflavone | nd | 4.9 | 39 | (9) |

1. Roland WSU, Buren L Van, Gruppen H, Driesse M, Gouka RJ, Smit G, Vincken J-P, van Buren L, Gruppen H, Driesse M, et al. Bitter taste receptor activation by flavonoids and isoflavonoids: modeled structural requirements for activation of hTAS2R14 and hTAS2R39. *J Agric Food Chem* (2013) **61**:10454–66. doi:10.1021/jf403387p

2. Narukawa M, Noga C, Ueno Y, Sato T, Misaka T, Watanabe T. Evaluation of the bitterness of green tea catechins by a cell-based assay with the human bitter taste receptor hTAS2R39. *Biochem Biophys Res Commun* (2011) **405**:620–625. doi:10.1016/j.bbrc.2011.01.079

3. Roland WSU, Vincken JP, Gouka RJ, Van Buren L, Gruppen H, Smit G. Soy isoflavones and other isoflavonoids activate the human bitter taste receptors hTAS2R14 and hTAS2R39. *J Agric Food Chem* (2011) **59**:11764–11771. doi:10.1021/jf202816u

4. Kohl S, Behrens M, Dunkel A, Hofmann T, Meyerhof W. Amino acids and peptides activate at least five members of the human bitter taste receptor family. *J Agric Food Chem* (2013) **61**:53–60. doi:10.1021/jf303146h

5. Meyerhof W, Batram C, Kuhn C, Brockhoff A, Chudoba E, Bufe B, Appendino G, Behrens M. The molecular receptive ranges of human TAS2R bitter taste receptors. *Chem Senses* (2009) **35**:157–170. doi:10.1093/chemse/bjp092

6. Lossow K, Hübner S, Roudnitzky N, Slack JP, Pollastro F, Behrens M, Meyerhof W. Comprehensive analysis of mouse bitter taste receptors reveals different molecular receptive ranges for orthologous receptors in mice and humans. *J Biol Chem* (2016) **291**:15358–15377. doi:10.1074/jbc.M116.718544

7. Soares S, Kohl S, Thalmann S, Mateus N, Meyerhof W, De Freitas V. Different Phenolic Compounds Activate Distinct Human Bitter Taste Receptors. *J Agric Food Chem* (2013) **61**:1525–1533. doi:10.1021/jf304198k

8. Ueno Y, Sakurai T, Okada S, Abe K, Misaka T. Human bitter taste receptors hTAS2R8 and hTAS2R39 with differential functions to recognize bitter peptides. *Biosci Biotechnol Biochem* (2011) **75**:1188–1190. doi:10.1271/bbb.100893

9. Schwiebert E, Wang Y, Xi R, Choma K, Streiff J, Flammer LJ, Rivers N, Ozdener MH, Margolskee RF, Christensen CM, et al. Inhibition of Bitter Taste from Oral Tenofovir Alafenamide. *Mol Pharmacol* (2021) **99**:319–327. doi:10.1124/molpharm.120.000071

10. Yamazaki T, Sagisaka M, Ikeda R, Nakamura T, Matsuda N, Ishii T, Nakayama T, Watanabe T. The human bitter taste receptor hTAS2R39 is the primary receptor for the bitterness of theaflavins. *Biosci Biotechnol Biochem* (2014) **78**:1753–1756. doi:10.1080/09168451.2014.930326

11. Morini G, Winnig M, Vennegeerts T, Borgonovo G, Bassoli A. Vanillin Activates Human Bitter Taste Receptors TAS2R14, TAS2R20, and TAS2R39. *Front Nutr* (2021) **8**:683627. doi:10.3389/fnut.2021.683627

12. Roland WSU, Gouka RJ, Gruppen H, Driesse M, van Buren L, Smit G, Vincken J-P. 6-methoxyflavanones as bitter taste receptor blockers for hTAS2R39. *PLoS One* (2014) **9**:1–10. doi:10.1371/journal.pone.0094451
